# Improving forecasts of individual ocean eddies using feature mapping

**DOI:** 10.1038/s41598-023-33465-9

**Published:** 2023-04-17

**Authors:** Tatiana Rykova

**Affiliations:** CSIRO, Environment, Hobart, TAS 7001 Australia

**Keywords:** Physical oceanography, Scientific data

## Abstract

Marine industries, war fighters, and world leaders demand accurate maps of ocean properties to underpin tactical and strategic decisions. Oceanographers have approached this challenge by borrowing mapping techniques from weather forecasters. However, compared to the atmosphere, the spatial scales of the ocean are small, and ocean properties are vastly under-sampled. Not surprisingly, despite decades of dedicated effort, the quality of maps of under-sea conditions remains poor. Feature mapping is a new approach to this problem that treats every ocean eddy individually. It strictly limits the influence of each observation to the oceanographic feature that it directly observes. Resulting maps are precise and realistic and may revolutionise ocean forecasting.

## Introduction

Oil and gas exploration, undersea warfare, marine conservation, and fisheries management share one thing in common. Motivated to reduce their risk and maximise their efficiency or advantage, they all base strategic and tactical decisions on estimates of subsurface ocean properties. Such estimates are typically delivered as ocean analyses and/or ocean forecasts—akin to prognostic weather charts and forecasts. These services are underpinned by ocean observations, constructed using data assimilation, and often enhanced by an ocean model.

Ocean observing is an expensive and resource-intensive undertaking^1–3^. Diligent exploitation of ocean observations is therefore important. Equally, production of gridded ocean products to underpin scientific research^4^ and to inform decision-making^5–7^ bares a responsibility to faithfully reproduce estimates of ocean properties as precisely as possible. With sustained development, the quality of gridded ocean products—including ocean analyses, reanalyses, and forecasts—is improving^8,9^. But accurate reproduction of subsurface ocean properties remains a challenge^10–13^. Some new approaches to this problem are emerging^14,15^, but most still conform to the same approach of combining all observations to construct global analyses.

**Figure 1:**
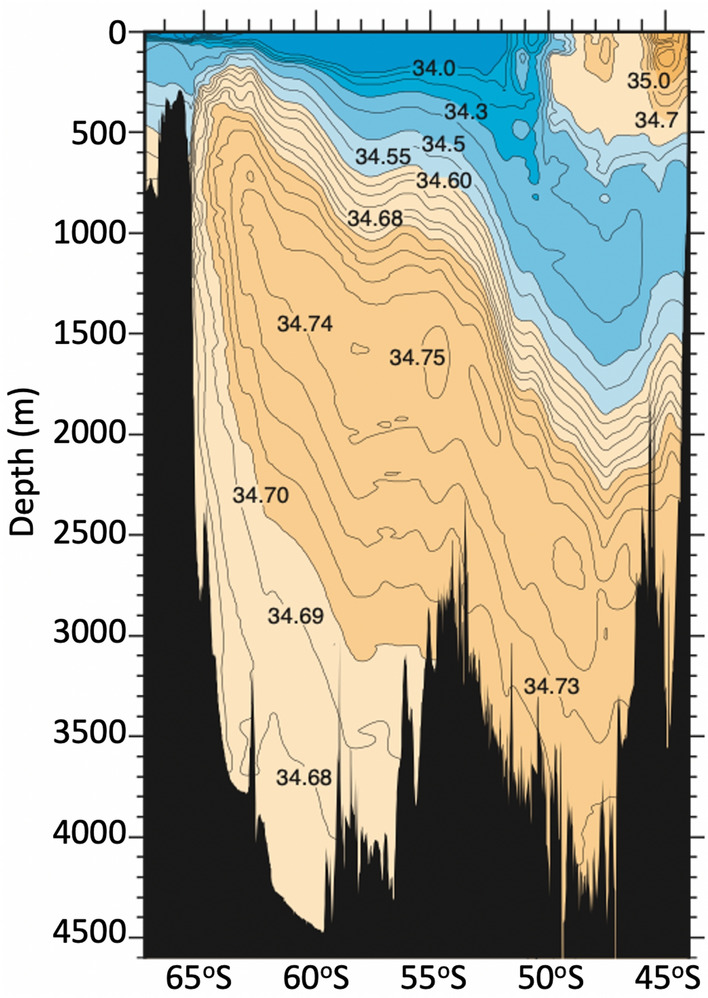
A demonstration of the level of detail included in WOCE sections—showing a latitude-depth section of salinity between Antarctica and Tasmania along WOCE line S3 (P12), in April 1994 (09AR9404_1; RSV Aurora Australis; Rintoul), downloaded from whp-atlas.ucsd.edu/pacific/s03/sections/ctd/1250/ctd_1250.htm.

Production of gridded fields using in situ observations from profiles of ocean properties has a long history—including construction of countless World Ocean Circulation Experiment (WOCE) sections^16^. WOCE sections are carefully constructed^17^ to preserve the detail of every measurement, with mis-fits to with-held data of less than 0.1 $^\circ$C for temperature, and less than 0.007 psu for salinity^17^. An example of a WOCE section in the Southern Ocean is presented in Fig. 1. This example shows a latitude-depth section of salinity with the dominant water masses, broadly denoted by different colours, and depths of different isohalines. WOCE sections often include small-amplitude, localised extrema. Figure 1 includes a local maximum—denoting the core of Circumpolar Deep Water—at about 1600 m depth at 55$^\circ$ S, with salinity of 0.01 psu higher than surrounding waters. Other local extrema are evident in this section, demonstrating the level of detail retained in WOCE sections to underpin oceanographic and climate research.

**Figure 2:**
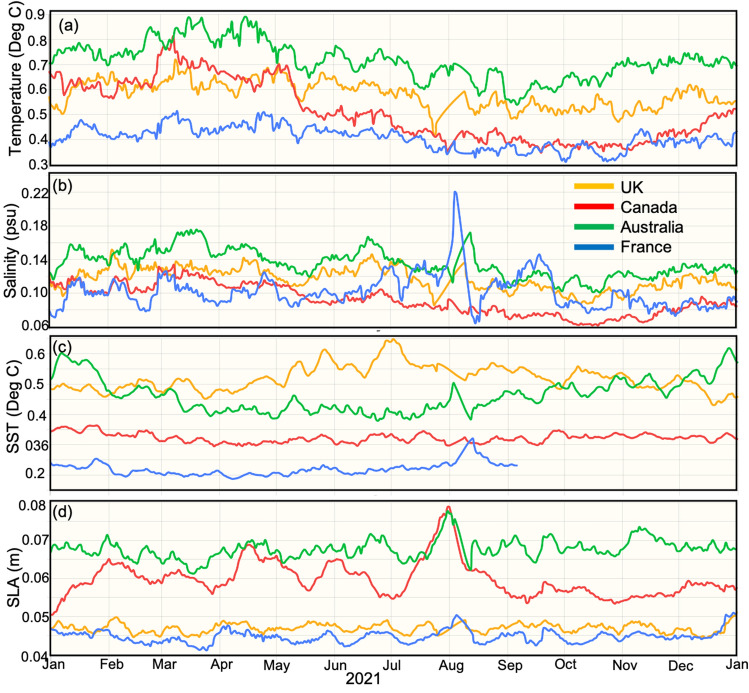
A demonstration of the precision of analyses from contemporary ocean forecast systems. Time series of RMSD between observed and analysed (**a**) subsurface temperature, (**b**) subsurface salinity, (**c**) surface temperature, and (**d**) sea-level anomaly for four different operational ocean forecast systems in the Australian region. The four systems include the operational short-range forecast systems from the UK^8^, Canada^41^, Australia^42^, and France^43^. These figures were accessed from https://seedragon.org on 13 December 2022, are were produced under OceanPredict (https://seedragon.org) to inter-compare “class 4” metrics between operational systems^11,44^.

By contrast to WOCE sections, the mis-fits between observations and analyses from high-resolution ocean forecast systems are large. Time series of the Root-Mean-Squared Differences (RMSD) between observed and analysed fields from four operational ocean forecast systems are shown in Fig. 2. The mis-fits between observed and analysed fields are 0.4–0.9 $^\circ$C for subsurface temperature (Fig. 2a), 0.06–0.16 psu for subsurface salinity (Fig. 2b), 0.2–0.6 $^\circ$C for sea surface temperature (SST; Fig. 2c), and 4–7 cm for sea level anomaly (SLA; Fig. 2d). These results are consistent with results from many other studies^10–12^. Measurement errors are 0.004 $^\circ$C for subsurface temperature and 0.01 psu for subsurface salinity from Argo^18^—the world’s most comprehensive in situ ocean observing platform^2^; and is 3–5 cm for SLA^19^ and 0.1–0.42 $^\circ$C for SST^20^. The mis-fits between subsurface observations and analyses from operational systems are more than 100 times greater than measurement error for temperature, and 5 times greater for salinity. By contrast, mis-fits between satellite observations and analyses are comparable to measurement error for both SLA and SST.

All data assimilation systems that underpin operational forecasts make explicit estimates of the amplitude of observation errors. These error estimates guide the degree to which analyses align with observations. Assumed observation errors are intentionally inflated for forecast systems to account for “representation error”^21,22^. Representation error is a real signal that cannot be reproduced on the target grid because the spatial scales of the signal are smaller than those resolved on the target grid. However, representation error is poorly understood, and so assimilating models tend to assume observation errors that are large, compared to measurement error, with assumed errors of the order of 0.5–1 $^\circ$C for temperature, and 0.2–0.5 psu for salinity^23,24^. This is one reason why ocean analyses produce fields in poor agreement with subsurface observations.

In this study, a complementary approach to data assimilation is proposed. Rather than combining all observations together to construct global analyses—the new approach is intended to refine gridded properties of the ocean—one feature at a time. This approach is called “Feature Mapping”, because the influence of observations is strictly limited to within the feature that they observe directly. This approach is a post-processing step that can complement the data assimilation systems that are widely employed by our community. This idea is borrowed from weather forecasting. In the 1970s, when numerical weather prediction (NWP) was immature, weather forecasts were poor. At that time, weather forecasters developed techniques called “post-processing”^25,26^, sometimes referred to as “forecast calibration”, that exploit archives of forecasts and their statistical relationships with observations^27–29^. An equivalent archive of observations and forecasts of the ocean is not readily available, and so a blind adoption of these techniques is out of reach. Instead, the developments here merely adopt the idea of refining gridded fields using observations.

Feature mapping does not replace existing data assimilation systems—but can be used as their complement, to refine gridded fields to better align with observations. Feature mapping applies oceanographic principles to adjust water mass properties within every observed feature, and to adjust the isopycnal depths of the feature. The resulting feature-mapped fields agree with observations to within the target precision, producing gridded fields with the accuracy of WOCE sections, but with the global coverage of contemporary ocean forecast systems.

## Methods

### Argo buddy analysis

Gridded products can only represent features that can be adequately resolved on the target grid. An analysis of properties on a 10-km resolution grid, for example, cannot represent any feature that varies spatially on scales shorter than 10 km (e.g., a sub-mesoscale eddy). To estimate the amplitude of these unresolved scales, we compare profiles of temperature and salinity from Argo floats that sample very close in time and space. This analysis estimates the precision to which analyses should “fit” analysed observations.

**Figure 3:**
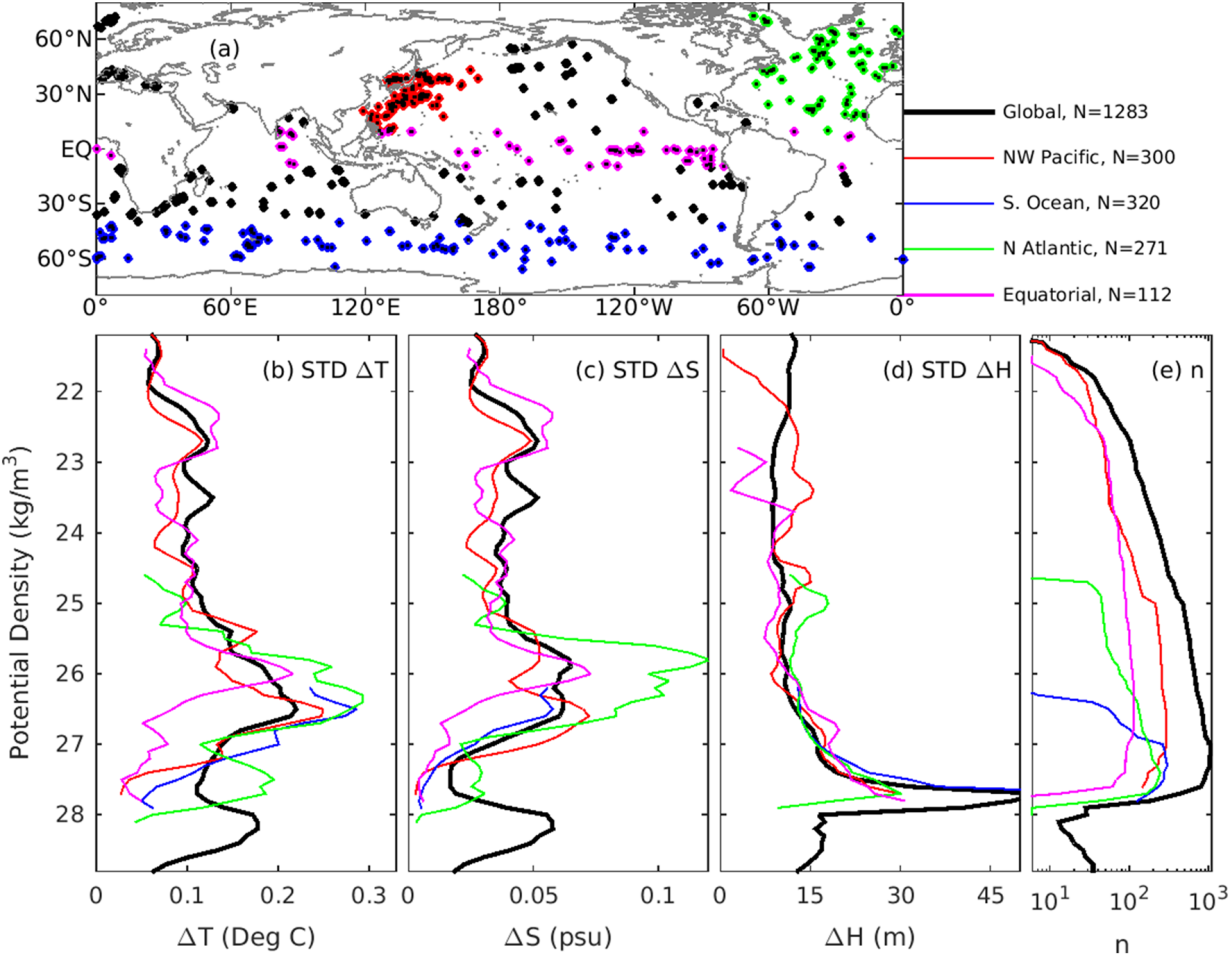
A demonstration of how close analyses should fit in situ observations for a 1/10$^\circ$-resolution grid. Analysis of Argo “buddy” floats that sample within 10 km and within 12 h of each other, showing (**a**) the location of each pair; and the standard deviation of the difference of (**b**) temperature (**c**) salinity, and (**d**) isopycnal depths, as a function of potential density; and (**e**) the number of observations, n, in each density range, plotted on a log scale. The colours in each panel are for different regions as denoted in the legend (also showing the number of buddy floats in each region, N). This analysis excludes the top 50 m, where water mass properties are often highly variable and where isopycnal depths are poorly defined.

Here, the amplitude of this unsolvable signal is estimated for temperature and salinity on a 1/10$^\circ$-resolution grid. To achieve this, we compare profiles from Argo floats when they sample within 10 km and 0.5 days of each other. For a 1/10$^\circ$-resolution grid, this would mean that these profiles are within the same grid box and on the same day. Results from this analysis are shown in Fig. 3. In total, there are 1283 profiles that have a “buddy” float that meets the required criterion. Using these buddy pairs, temperature and salinity is projected onto potential density surfaces, and the depth of isopycnals, for potential density in the range of 21–29 kg/m$^{-3}$, are calculated in bins of 0.1 kg/m$^{-3}$ (21, 21.1, 21.2, etc). No single profile spans this range, so we only populate values within the range of each profile. In density space, we then compare buddies, and calculate the standard deviation of the differences of temperature, salinity, and isopycnal depth. We present these standard deviations as a function of potential density in Fig. 3, along with a map showing the location of each buddy pair, and the number of observations for each density value. We present statistics from the entire globe, and separated by region in Fig. 3.

Based on this analysis (Fig. 3), it seems that a reasonable target for how close a gridded field should “fit” in situ observations, on a 1/10$^\circ$-resolution grid, should be 0.1 $^\circ$C for temperature, and 0.04 psu for salinity. This is the typical amplitude of differences between buddy floats on sub-grid-scales of a 1/10$^\circ$-resolution grid. Recall that WOCE sections typically produce gridded fields within this range of precision (Fig. 1), but that widely-used global analysis systems do not (Fig. 2).

### Feature mapping

#### Methodology

Feature mapping is a post-processing method that refines gridded fields of temperature and salinity to better align with observations. This method is applied to one eddy at a time, and is applied to eddies that are directly sampled by an Argo float, or by a platform that measures temperature and salinity over a significant depth range (e.g., a glider or shipboard conductivity-temperature depth profile). Argo floats usually sample between the surface and 2000 m depth, covering the most variable parts of the water column, and so are ideal for this application. Feature mapping compares gridded and observed profiles of temperature and salinity, in density space, and uses the differences to correct the water properties throughout each sampled eddy. Similarly, feature mapping compares gridded and observed isopycnal depths, and uses the differences to correct isopycnal depths throughout each eddy. Feature mapping assumes that the mis-fits between the gridded and observed fields are applicable throughout the entire eddy—or feature—and can therefore be used to adjust, or correct, the feature’s properties in three dimensions.

Feature mapping requires a background field of gridded temperature $T^{BG}(x,y,z)$, salinity $S^{BG}(x,y,z)$, and sea-level anomaly $SLA^{BG}(x,y)$, where *x*, *y*, and *z* are the zonal, meridional, and vertical directions respectively, and the super-script, *BG*, denotes background. The gridded fields should be from an analysis—meaning that they should be constructed using observations—produced by a data-assimilating model^24^, an objective analysis system^30^, or similar^31^. It is important that $SLA^{BG}$ is realistic, with eddies in approximately the right location, and of approximately the right size and shape. Such fields are readily accessible, as noted above, with reference to Fig. 2d.

To apply feature mapping, a candidate eddy that has been directly observed by a profiling platform (e.g., an Argo float) is first identified. The boundary of the eddy is defined as the outer-most contour of $SLA^{BG}$ that encompasses a single local extrema—a local maximum for an anticyclonic eddy, or a local minimum for a cyclonic eddy. Multiple local extrema are not permitted, to ensure that the candidate eddy is a well-defined, isolated, and coherent feature. This is necessary because feature mapping assumes that the mis-fits between observed and gridded fields within an eddy are representative of errors throughout the entire eddy. This assumption is only reasonable for coherent, well-defined features.

**Figure 4:**
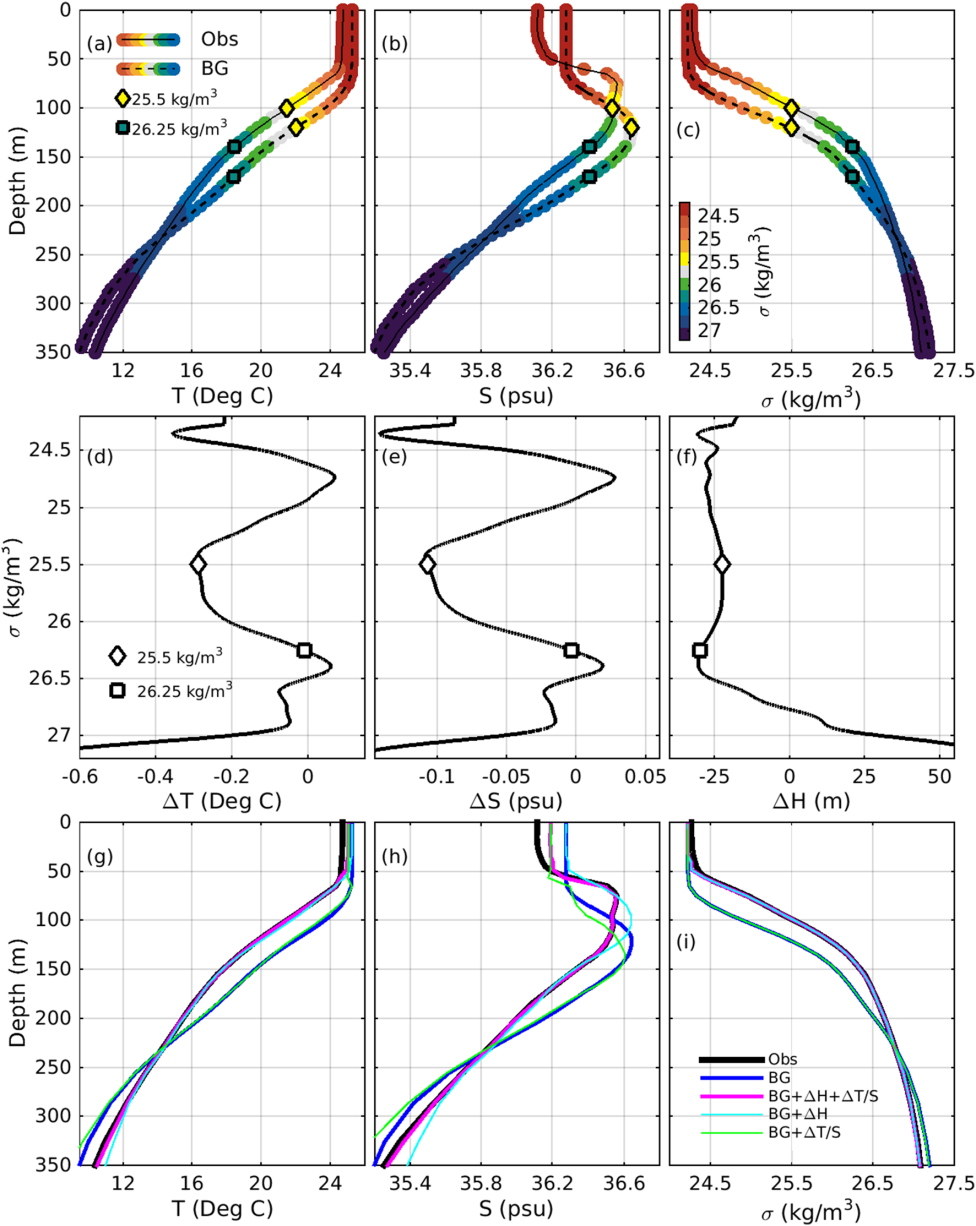
Profiles of (**a**) temperature, (**b**) salinity, and (**c**) density from observations and a background field; the difference between the observed and background, (**d**) temperature, (**e**) salinity, and (**f**) isopycnal depth; and profiles of (**g**) temperature, (**h**) salinity, and (**i**) density at each stage of feature mapping. In panels (**a–c**), profiles are plotted as a function of depth; and in panels (**d–f**), profiles are plotted as a function of potential density. In panels (**a–c**) the coloured dots along each profile denote the potential density. The colorbar for density is shown in panel (**c**). The diamonds (squares) in each panel highlight the value of each profile where the density is at 25.5 (26.25) kg/m$^3$. The profiles in (**d–f**) are examples of the values of the LUT used for feature mapping: (**d**) $\Delta T^{LUT}$, (**e**) $\Delta S^{LUT}$, and (**f**) $\Delta H^{LUT}$, and the density vector for those panels are $\sigma ^{LUT}$.

Feature mapping includes three separate calculations: construction of a two-dimensional mask, corrections to water mass properties, and corrections to isopycnal depths. To quantify the required adjustments to water mass properties and isopycnal depths, the differences between the observed and gridded temperature, salinity, and isopycnal depths are calculated at the location of the observed profile, and are assembled in a look-up table (LUT). Examples of such profiles are presented in Fig. 4a–c. The differences are calculated as a function of potential density (Fig. 4d–f) so that the adjustments to the eddy’s properties can be performed in density space. This approach is taken because in the ocean interior, water properties tend to be more coherent along density surfaces, with water routinely moving along isopycnal layers without opposition from buoyancy and without significant mixing^32^.

A vector of density values, $\sigma ^{LUT}$ is defined, spanning the full range of densities in the background field, with increments of 0.01 kg/m$^3$. The choice of the density increment in this vector is a trade-off between efficiency (too small and the calculations become slow) and accuracy (too large, and the feature mapping becomes imprecise in regimes of low stratification). For each value of the observed density (denoted by the colours along the profiles presented in Fig. 4a–c), the difference between the observed temperature and gridded temperature (interpolated to the observed location and depths) is calculated, and is objectively mapped onto the vector $\sigma ^{LUT}$ (Fig. 4b) to produce $\Delta T^{LUT}$. This one-dimensional objective analysis^30^ assumes an *e*-folding decorrelation scale of 0.5 kg/m$^3$ (this scale is analogous to a length-scale used for conventional objective analysis, but is expressed here as density, because the mapping is performed in density space, not in physical space). An example of values in $\Delta T^{LUT}$ is presented in Fig. 4d. An equivalent analysis is performed for salinity and isopycnal depths to assemble the mis-fits for all three variables to produce $\Delta T^{LUT}$, $\Delta S^{LUT}$, and $\Delta H^{LUT}$, for every value of $\sigma ^{LUT}$ (Fig. 4d–f). The objective analysis step used to populate the LUT requires explicit estimates of the observation and background error variance, or standard deviation, to avoid over-fitting the observations^30^. The assumed observation error standard deviations are guided by the Argo buddy analysis (Fig. 3), and are assumed to be 0.1 $^\circ$C for temperature, 0.04 psu for salinity, and 10 m for isopycnal depths. Similarly, the assumed background error standard deviations are guided by the errors quantified in Fig. 2a,b, and are assumed to be 0.5 $^\circ$C for temperature, and 0.1 psu for salinity. We note that background errors for temperature and salinity are several times larger than the observation errors. An appropriate error for background isopycnal depth is not immediately evident from Fig. 2. Here, the background error standard deviation for isopycnal depths is assumed to be 30 m. This is a few times larger than the assumed observation error standard deviation, consistent with the relative errors assumed for the observed and background temperature and salinity (we have tested the system with larger background errors—up to ten times greater than observation errors—and find that assuming larger background errors sometimes over-fits the observations, generating occasional density inversions).

With the LUT assembled, the correction of water mass properties is straightforward. For every grid point, in three dimensions within the eddy, the background density is calculated, and the corresponding value for $\Delta T^{LUT}$ and $\Delta S^{LUT}$ is identified and used to construct a three-dimensional adjustment to temperature and salinity, $\Delta T^{water\,mass}(x,y,z)$ and $\Delta S^{water\,mass}(x,y,z)$, where the super-script indicates an adjustment to correct water mass properties. Consider the example points highlighted in Fig. 4, where the diamonds denote values where density is 25.5 kg/m$^3$, and the squares denote values where density is 26.25 kg/m$^3$. For the example where density is 25.5 kg/m$^3$, the observed temperature is slightly colder than the background temperature (Fig. 4a), resulting in a $\Delta T^{LUT}$ of about − 0.3 $^\circ$C (Fig. 4d). Similarly, where density is 25.5 kg/m$^3$, the observed salinity is slightly fresher than the background salinity (Fig. 4b), resulting in a $\Delta S^{LUT}$ of about − 0.11 psu (Fig. 4e). For the other example, where density is 26.25 kg/m$^3$, denoted by the squares, the observed and background temperature and salinity are almost the same (Fig. 4a,b), resulting values for $\Delta T^{LUT}$ and $\Delta S^{LUT}$ of close to zero—meaning that the water mass properties on this density surface are already well-aligned with observations, and require no significant refinement.

The impact of the water mass adjustments for the example referred to above, is presented in Fig. 4g–i (compare the black, blue, and green profiles). Adding the water mass adjustment to the background temperature is barely evident (the green line overlays blue for most depths shown in Fig. 4g). Adding the water mass adjustment to the background salinity is only clearly evident between about 75–200 m depth (Fig. 4h). This is in the vicinity of the 25.5 kg/m$^3$ isopycnal that is highlighted by the diamonds in Fig. 4b,e, and is where $\Delta S$ is largest. Adding the water mass adjustments to the background density has no impact at almost all depths, with the green line entirely overlaying the blue line in Fig. 4i. The only exception is at the base of the vertically well-mixed layer, at about 50 m depth. In this regime, where the vertical gradient in density is large, there is insufficient resolution in $\sigma ^{LUT}$ to precisely represent adjustments of the water mass properties. This limitation warrants further investigation.

Again, using the LUT, a three-dimensional field of corrections to isopycnal depths is assembled. For every grid point the background density is calculated, and the corresponding values for $\Delta H^{LUT}$ are identified, and used to construct $\Delta H(x,y,z)$. An example of entries for $\Delta H^{LUT}$ is presented in Fig. 4f. For this example, the isopycnal depths of the background profile are about 25 m deeper than the observed profile between the surface and about 200 m depth (Fig. 4c), corresponding to a density range of about 24.3–26.5 kg/m$^3$ (Fig. 4f). To align the background densities with the observed profile, all properties over this density range must be shoaled by about 25 m. For densities of about 26.75 kg/m$^3$, no adjustment of isopycnal depths is required. But for densities close to 27 kg/m$^3$, isopycnals need to be deepened by about 25 m (Fig. 4f). This step requires some care to ensure that the profiles of $\Delta H$ do not introduce any values that make density unstable.

To translate the required corrections of isopycnal depths ($\Delta H(x,y,z)$) to adjustments of temperature and salinity, $\Delta T^{iso}(x,y,z)$ and $\Delta S^{iso}(x,y,z)$, where the super-script indicates an adjustment to isopycnal depths, a procedure of “vertical heaving” is applied. This procedure has long been used for data assimilation, exploiting the fact that it permits profiles to be deepened or shoaled, without changing the water mass properties^33,34^. This procedure is one-dimensional—translating each vertical profile of $\Delta H$ into profiles of $\Delta T^{iso}$ and $\Delta S^{iso}$. So, for a single profile of temperature at the *ith* horizontal grid point, we can express the adjustment to the background temperature profile as:

$$\begin{aligned} \Delta T^{iso}_i(z) = T^{BG}_i \big ( z+\Delta H_i(z) \big ) - T^{BG}_i (z), \end{aligned}$$

where the subscripts *i* indicate the *ith* horizontal grid point, at $(x,y)=(x_i,y_i)$. Here, $\Delta H_i(z)$ is the profile of vertical displacements at the *ith* horizontal grid point, and expresses a “heave”, or vertical displacement, of the background temperature. This is achieved, in practice, by interpolating between gridded levels, where the target depth levels have been adjusted by $\Delta H_i(z)$; effectively displacing depth levels from *z*, to $z+\Delta H_i(z)$. This step involves extrapolation of properties near the surface and near the seafloor. This is achieved by extending the shallowest (deepest) value of temperature or salinity to the surface (sea floor) when a profile is being deepened (shoaled). Every horizontal grid point within the eddy is processed separately, using the corresponding profile of $\Delta H$ for each location. An equivalent calculation is performed to produce $\Delta S^{iso}(x,y,z)$. This procedure merely shifts the profiles of temperature and salinity vertically. For grid points at which $\Delta H$ is negative, the vertical profile is shifted upwards (shoaled), and for grid points at which $\Delta H$ is positive, the vertical profile is shifted downwards (deepened). For a case where a profile of $\Delta H$ is one value over the entire water column, the profile is either shoaled or deepened, and the vertical gradients of the temperature and salinity, and the thickness of all density layers, are preserved. Because temperature and salinity are heaved together, the water mass properties, before and after the adjustment, remain unchanged.

Sometimes, the profile of $\Delta H$ is negative at lower densities (shallower depths), and positive at higher densities (deeper depths). This is the case for the example presented in Fig. 4c,f. This means that the layers in between these different regimes will be thickened, which is the case between about 26.5 and 27 kg/m$^3$ for the example in Fig. 4f. Conversely, suppose the profile of $\Delta H$ is positive at shallower depths, and negative at deeper depths—then the layers in between the regimes will become thinner. To visualise this, picture a vertically-aligned concertina. Depending on how the profile of $\Delta H$ changes over depth, the concertina might compress at some levels, and expand at others—resulting in thinning density layers at some depths and thickening density layers at other depths. When $\Delta T^{iso}(x,y,z)$ and $\Delta S^{iso}(x,y,z)$ are added to the background field, the resulting adjusted profiles align closely with the observed density profiles, but the water mass properties on each density surface are unaltered^33,34^.

The impact of the isopycnal adjustments for the example referred to above is also presented in Fig. 4g–i (compare the black, blue, and cyan profiles). Adding the isopycnal adjustment to the background temperature is clearly evident, with the adjusted profile overlaying the observed profile for most of the depths shown (Fig. 4g)—the only exception is below 300 m. Adding the isopycnal adjustment to the background salinity is most evident around 100 m depth, and below 250 m depth (Fig. 4h). Inspection of the background salinity, with (cyan) and without (blue) the isopycnal adjustment, clearly shows how the salinity profile is “heaved” vertically—shoaling at shallow depths, and deepened at deeper depths (Fig. 4h). Adding the isopycnal adjustment to the background density aligns the adjusted profile closely with the observed profile (Fig. 4i).

Because all of the calculations described above are performed in density space, the order in which the adjustments are calculated makes no difference. To understand this, consider again the example points highlighted by the diamonds and squares in Fig. 4. For both examples, the isopycnals are required to be shoaled by about 25 m. Here, we can see that the adjustments to isopycnals are in the vertical direction, in the framework of Fig. 4c—shoaling the background profiles over the top 200 m, or so, where density ranges from 24.3 to 26.5 kg/m$^3$; making no change around 250 m depth; and deepening isopycnals below that. This can be seen by the difference in depths of the diamonds and squares along the observed and background profiles in Fig. 4c; and is clearly evident in the adjusted salinity profiles in Fig. 4h, as noted above. Furthermore, we can see that the adjustments to water mass properties on density surfaces are orthogonal to the isopycnal adjustments—cooling and freshening the water at 25.5 kg/m$^3$; and making no change at 26.25 kg/m$^3$, where the observed and background water mass properties are already aligned. In the framework of Fig. 4, the isopycnal adjustments shift all profiles vertically; and the water mass adjustments shift the temperature and salinity profiles horizontally. As noted, the order in which the adjustments are applied makes no difference. The adjustments to isopycnal depths align the density profiles, without changing the water mass properties at each density level; and the adjustments to temperature and salinity on density surfaces align the water mass properties, without changing the isopycnal depths.

Feature-mapped fields are produced by adding adjustments for both the water mass properties and isopycnal depths to the background field. This is demonstrated for the individual calculations described above in Fig. 4g–i for profiles at the observation location. To restrict the changes to within the feature, when the adjustments are applied in three dimensions—to the entire eddy, we define a two-dimensional mask using $SLA^{BG}(x,y)$. We regard the “core” of the eddy—where we wish to apply the full adjustments—to be the area for which $|SLA^{BG} |$ exceeds 60% of the maximum $|SLA^{BG} |$ in the eddy. The value of 60% is chosen here because it seems to produce good results—but this parameter can be tuned for any application. We define $SLA_{core} = 0.6 |SLA^{BG}_{extrema} |$, where the subscript *extrema* denotes the local extrema (a maximum in an anticyclonic eddy; and a minimum in a cyclonic eddy). The boundary of the eddy is here defined as $SLA_{boundary}$, the absolute value of the outer-most contour of $SLA^{BG}$ that encompasses a single local extrema. We have also tested the system using $0.2 |SLA^{BG}_{extrema} |$ to define the boundary, but the outer-most SLA contour is a more objective criterion. The two-dimensional mask, $\rho (x,y)$, is given by:

$$\begin{aligned} \rho (x,y) = f(SLA^{BG}) = \left\{ \begin{array}{ll} 1 &{} \quad |SLA^{BG} |> SLA_{core}\\ \frac{ |SLA^{BG} |- SLA_{boundary}}{SLA_{core} - SLA_{boundary}} &{} \quad SLA_{boundary} \le |SLA^{BG} |\le SLA_{core} \\ 0 &{} \quad |SLA^{BG} |< SLA_{boundary} \end{array}. \right. \end{aligned}$$

This produces a mask that is one within the core of the eddy; zero outside of the eddy; and smoothly changing between the core region and the boundary with a shape that is derived from $SLA^{BG}$. With the mask defined, feature-mapped fields of temperature and salinity, $T^{FM}(x,y,z)$ and $S^{FM}(x,y,z)$, are produced, where the super-script *FM* denotes feature mapping; by adding the adjustments to water mass properties and isopycnal depths to the background field, and localising the adjustments using the mask. The feature-mapped fields are therefore given by:

1 $$\begin{aligned} T^{FM}(x,y,z) = T^{BG}(x,y,z) + \rho (x,y) \circ \Delta T^{water\,mass} (x,y,z) + \rho (x,y) \circ \Delta T^{iso} (x,y,z), \end{aligned}$$

and similarly for salinity, where the open circle, $\circ$, denotes a Schur product^35^ (an element-by-element multiplication) that is applied for each depth level. Application of the mask strictly limits the adjustments to within the eddy, and produces a seamless updated field, with no sharp boundaries between the feature-mapped eddy, and the original, un-adjusted background field in the surrounding water.

The impact of the water mass and isopycnal adjustments together for the example referred to throughout this section, is also presented in Fig. 4g–i (compare the black, blue, and magenta profiles). Together, the adjusted, feature-mapped profiles align with the observed profiles for temperature, salinity, and density, over all interior depths (below about 50 m depth). In Fig. 4i, it is difficult to see the profile of density for the feature-mapped field with both corrections applied (magenta), because it is overlaid by the profile with $\Delta H$ added (cyan). The only depth range for which the feature-mapped fields do not align so well with observations is near the surface, where the properties are vertically well-mixed. In this regime, as noted above, the calculations in density space that are fundamental to feature mapping are not ideal. That is because the small range of densities of the surface mixed layer in the background and observed fields are not well-resolved by $\sigma ^{LUT}$.

The feature-mapped fields align with observations—with more precise water mass properties, and with isopycnal depths that match observations in the ocean interior. While the properties of the feature-mapped fields are refined, the spatial structure (the size and shape of the eddy) of the feature being post-processed is retained, and no properties outside of the feature are altered.

#### Limitations

Many post-processing methods^25,26^ and some data assimilation systems^36,37^ use observations more than once to refine analyses. Similarly, a profile used for feature mapping may have already been used to construct the background field. If the background field fits the profile well, then adjustments from (1) will be small (possibly zero if the background perfectly matches the observed profile).

As noted, feature mapping does not attempt to refine the size and shape of the eddy being post-processed. But because the mask is a function of $SLA^{BG}$, the accuracy of the $SLA^{BG}$ is important. If $SLA^{BG}$ is poor, the feature-mapped field will also be poor. But as noted, realistic SLA analyses are readily produced.

Feature mapping also requires sufficient overlap between the observed density range and the background density range (for construction of the LUT). If there is limited, or no overlap, feature mapping may be unfeasible. This can happen at high latitudes, where the density range is often small, and where some gridded products may have a significant bias. For most of the ocean, this is not the case, and feature mapping offers an efficient tool for reliably post-processing gridded fields.

## Results

### Assessment using Argo

**Figure 5:**
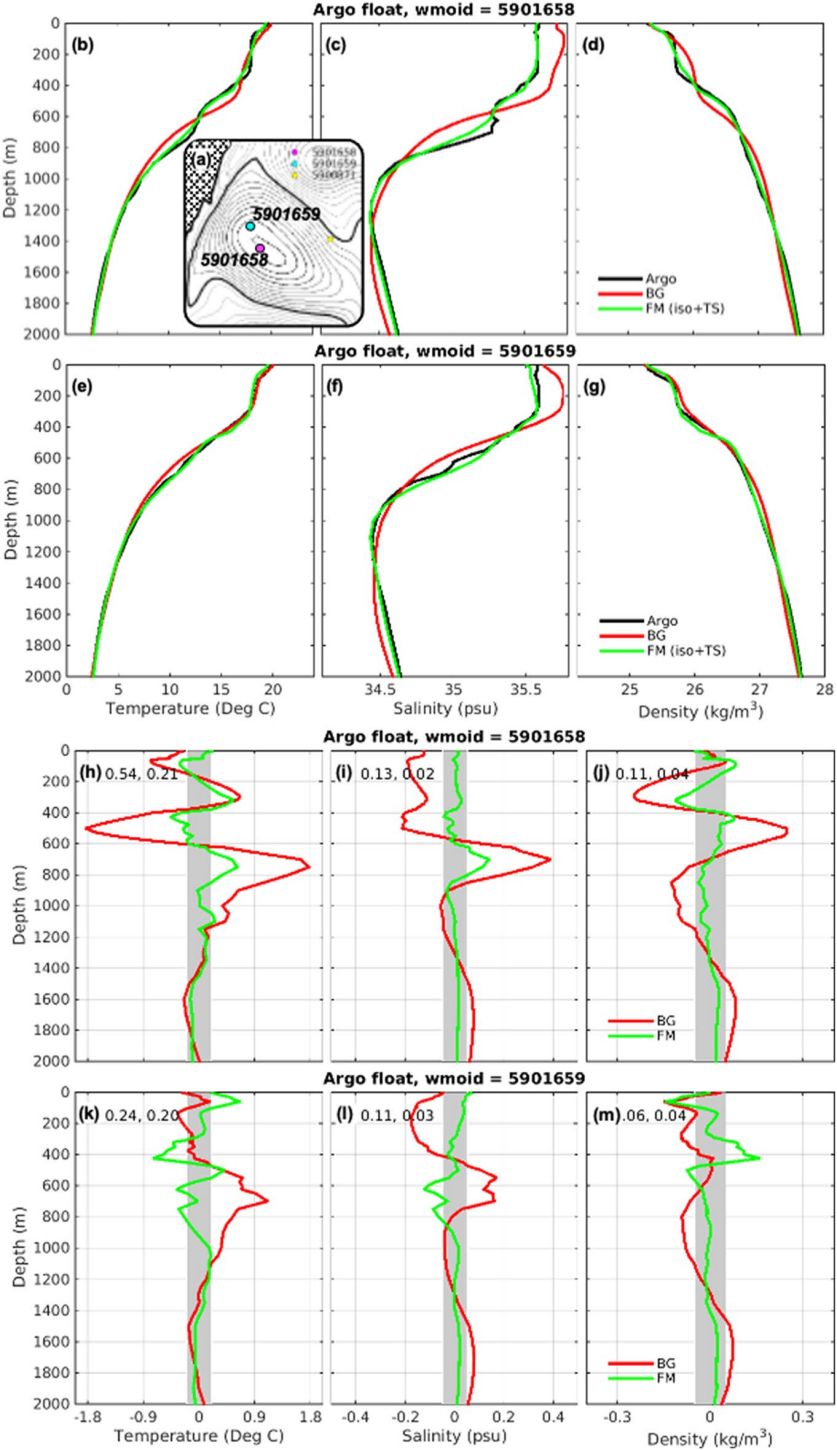
A demonstration of feature mapping, using independent Argo profiles for validation. Profiles of (**b–g**) observed (black), background (red) and feature-mapped (green) temperature, salinity, and potential density; and (**h–m**) mis-fits between observed profiles and background (red) and feature-mapped (green) profiles; within an eddy off south-eastern Australia. The RMSD between the observed and BG and observed and FM profiles are reported in panels (**h–m**). The grey bands denote the target mis-fits, based on the Argo buddy analysis. Here, two Argo floats sample the same anticyclonic eddy—their locations are denoted in panel (**a**), overlaying a map of gridded SLA (black contours are positive; grey contours are negative; bold contour is zero; hatched is land). Profiles from float 5901658 are used to adjust the eddy properties, and profiles from float 5901659 are used here as independent validation.

To demonstrate how feature mapping works, we apply it to an anticyclonic eddy off south-eastern Australia (Fig. 5) for a time when two Argo floats sampled the same eddy at approximately the same time. This example is non-trivial, and includes a complex multi-layer structure that resulted from the merging of two eddies^38^. We use profiles from one float to perform the feature mapping, and profiles from a second—independent—float to validate the feature-mapped fields. Here, we use background fields from a global ocean reanalysis^24^. For this example there are large differences between the observed and background profiles (Fig. 5h–m; red lines).

By contrast, the feature-mapped fields (Fig. 5; green lines) are in good agreement with observations (black lines), with mis-fits that mostly fall within the target precision identified in the Argo buddy analysis (shaded area in Fig. 5h–m). Both the profile used for feature mapping (from float 5901658) and the independent profile (from float 5901659) are well-aligned with observations.

### Assessment using another model

**Figure 6:**
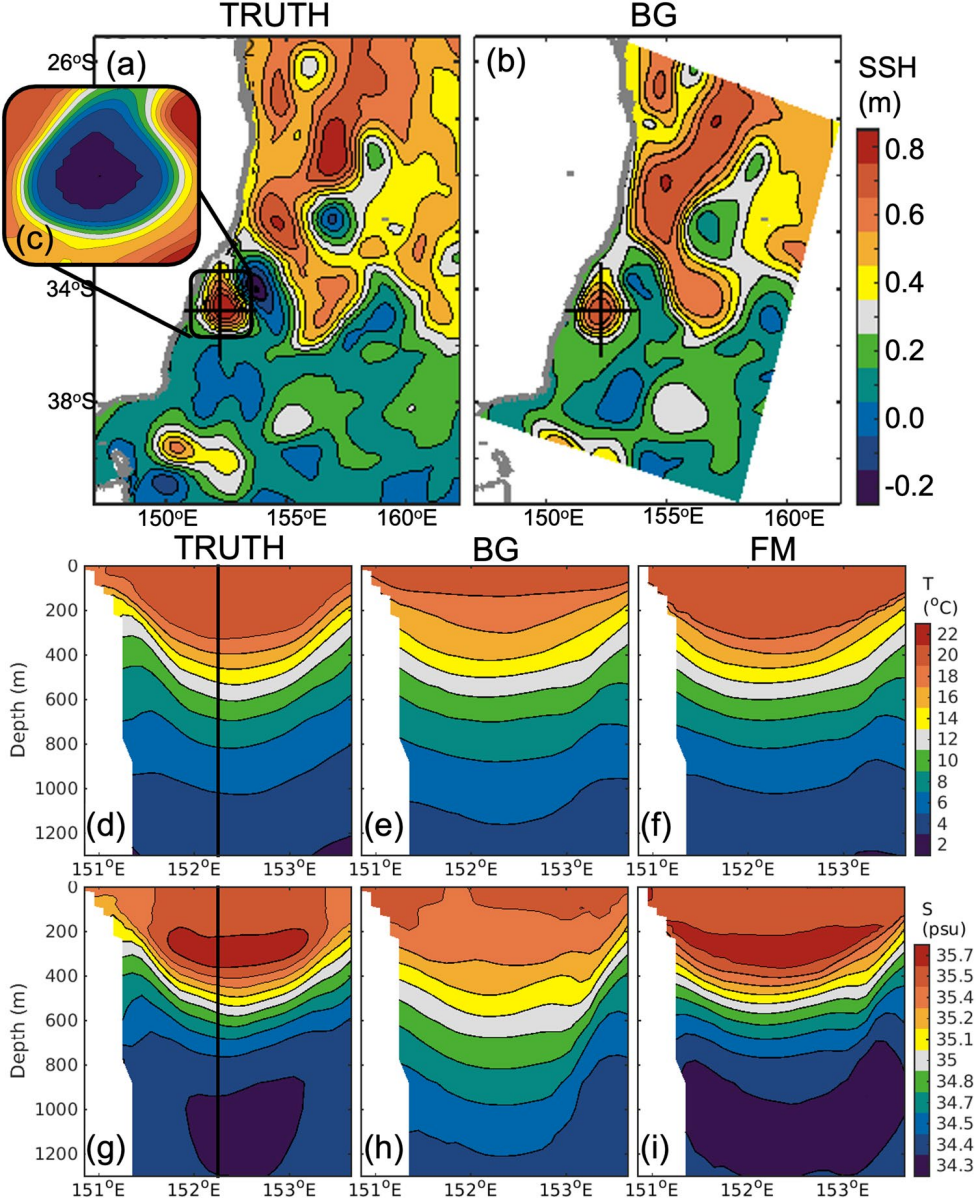
A demonstration of feature mapping, using fields from an independent model validation. (**a,b**) Gridded SLA from two different models, one is treated as the true ocean, and one is treated as the background. (**c**) Two-dimensional mask used to define the feature. Longitude-depth sections of (**d–f**) temperature and (**g–i**) salinity, showing fields from the (**d,g**) truth (the vertical black line denotes the location of the “observed” profile used for feature-mapping), (**e,h**) background (BG), and (**f,i**) feature-mapped (FM) fields.

Another way to assess the new method is to use fields from two different models, with one treated as the true ocean (hereafter, the truth), and a second treated as the background—so-called twin experiments^39^. Here, gridded fields from a 1/10$^\circ$-resolution global ocean reanalysis^24^ are used to represent the truth, and fields from a 4 km-resolution regional ocean reanalysis^40^ are used as the background. First, the gridded SLA field from the background is used to identify the boundary of the feature of interest—here, a large anticyclonic eddy adjacent to the coast off south-eastern Australia (Fig. 6a–c). Next, a profile of temperature and salinity from the truth is sampled, and uncorrelated noise, with a standard deviation of 0.1 $^\circ$C and 0.04 psu, is added to the “observed” profiles. Feature mapping is applied, and the background field is adjusted using only a single profile from the truth. A comparison of the true, background, and feature-mapped temperature and salinity fields is presented in Fig. 6d–i. For this example, the true and background fields are quite different. For example, the true field includes a deep thermocline and halocline, a subsurface salinity maximum between 200 and 400 m depth, and a subsurface salinity maximum below 800 m depth (Fig. 6d,g). These characteristics are not evident in the background field (Fig. 6e,h). Using just one profile from the truth, the salient elements of the true field are reproduced in the feature-mapped fields (Fig. 6f,i). The refined field does not perfectly match the true field—but differences are smaller—and are mostly less than the target precision of 0.1 $^\circ$C for temperature and 0.04 psu for salinity.

## Discussion

Ocean analysis and forecast systems have largely adopted methods developed for NWP. However, the length-scales of the ocean are much shorter than the atmosphere, and the ocean is grossly under-sampled compared to the atmosphere. As a result, the precision of ocean analyses is generally poor, producing analyses that do not fit observations as accurately as many applications require. Drawing on ideas of post-processing from NWP, and applying oceanographic analysis techniques, a new method for refining oceanic features that are directly observed is proposed. Instead of adjusting the properties of every grid point in an analysis using all observations together, we strictly limit the influence of observations to well-defined features that are directly observed. The method is demonstrated here to refine the analysed properties of eddies in the Tasman Sea. Using just a single profile of temperature and salinity from within an eddy, feature mapping employs oceanographic principles to adjust the water mass properties—rendering the analysed water masses realistic; and adjusts the isopycnal depths, to produce a refined gridded product that is well-aligned with observations.

Feature mapping exploits a different approach to conventional analysis systems. Conventional methods produce analyses of properties for every grid point by projecting observations from nearby observations onto each grid point. This can lead to information from one feature “contaminating” analyses in other features. By contrast, feature mapping is implemented by first identifying all the clearly-defined features of the ocean. These are considered candidates for feature mapping. Then those features that are sampled by an Argo float are identified, and their properties are refined. In practice, an Argo array of 3500 floats reports about 350 profiles each day. Of these 350 profiles, an average of 40–50 are within well-defined eddies. Over a 10-day period, this means that the properties of up to 500 eddies can be refined using feature mapping. This only represents about 2.5% of the area of the ocean, but it includes the most energetic features of the ocean circulation. Over time, as floats sample different features, near-global coverage can be achieved and the properties of ocean analyses can be refined—one eddy at a time.

Feature mapping is computationally efficient, and can be used to post-process gridded fields from any analysis, reanalysis, or forecast product, with no special information about how each product is produced. Feature mapping may deliver to the ocean forecasting community, what post-processing, or forecast calibration, delivered to NWP—uplifting the quality of analyses and forecasts to meet the needs of end-users.

## Data Availability

BRAN2020 data, analysed during the current study, are available on Australia’s National Computational Infrastructure’s (NCI’s) THREDDS server, at https://dapds00.nci.org.au/thredds/catalog/gb6/BRAN/BRAN2020/catalog.html. Argo data, analysed during the current study were collected and made freely available by the International Argo Program (https://argo.ucsd.edu, https://www.ocean-ops.org). The datasets produced during the current study can be made available from the corresponding author on reasonable request.
